# Prognostic value of combining preoperative immune-inflammatory-nutritional index and tumor biomarkers in gastric cancer patients undergoing radical resection

**DOI:** 10.3389/fnut.2025.1562202

**Published:** 2025-06-10

**Authors:** Yongtao Du, Yunlong Li, Zhaobang Tan, Jiawei Song, Yu Jiang, Shuai Liu, Yajie Guo, Yihuan Qiao, Jun Zhu, Shisen Li, Jipeng Li

**Affiliations:** ^1^Department of Digestive Surgery, The First Affiliated Hospital of Air Force Medical University, Xi’an, Shaanxi, China; ^2^School of Clinical Medicine, Xi'an Medical University, Xi'an, Shaanxi, China; ^3^Department of General Surgery, The Southern Theater Air Force Hospital, Guangzhou, China; ^4^Department of Experiment Surgery, Xijing Hospital, The First Affiliated Hospital of Air Force Medical University, Xi'an, China

**Keywords:** gastric cancer, cachexia, Prognostic Nutritional Index, Systemic Immune Inflammation Index, prognostic model, nomogram, calibration curve

## Abstract

**Background:**

Gastric cancer (GC) patients frequently face the debilitating comorbidity of malignant cachexia, a condition that consistently forecasts a dismal prognosis. Early diagnosis of cachexia and timely prediction of survival outcomes are essential for them. Here, we aimed to construct an immune-inflammatory-nutritional-tumor-marker (IINTM) prognostic score for GC, and further scrutinize its clinical relevance in early forecasting the cachexia.

**Method:**

A total of 1,101 GC patients underwent curative surgical were incorporated in our study, and they were evaluated by the Computed Tomography (CT) of skeletal muscle mass at third lumbar spine plane levels (SMI-L3). Using restricted cubic spline (RCS) analysis, we examined associations between prognosis and nutritional indices, including the Prognostic Nutritional Index (PNI) and Systemic Immune-Inflammation Index (SII). The IINTM score was constructed by the multivariate Cox analysis and evaluated by the Receiver Operating Characteristic (ROC) and area under the ROC (AUC).

**Results:**

We identified striking discrepancies in immunonutrition profiles and prognoses between cachexia and non-cachexia GC patients. Patients with cachexia had worse prognosis and lower SMI-L3 scores than those without cachexia. The IINTM score, incorporating PNI, SII, body mass index (BMI), NRS2002, serum albumin, platelet, D-dimer, CEA, and CA199, exhibited a high concordance index (C-index) of 0.784, underscoring its robust predictive efficacy. Most crucially, IINTM score demonstrated substantial diagnostic value for cachexia, with an AUC of 0.858, denoting its high degree of accuracy.

**Conclusion:**

The IINTM score could be a reliable tool and precisely predict the cachexia and prognosis for GC patients. Our findings provide novel insights into the role of immune-inflammatory-nutrition, tumor marker and cachexia in GC patients.

## 1 Introduction

Gastric cancer (GC) is one of the most common digestive tract malignancies worldwide, and its incidence and mortality are the fifth and fourth among malignancies in the world (1). Abundant reports revealed that 60–80% of late-stage cancer patients would experience cachexia. Specially, the incidence of cachexia in gastrointestinal cancer is as high as 87% (2), which is strongly associated with serious consumption of the body’s nutrient reserves by the gastrointestinal cancer. This devastating condition is characterized by muscle depletion of skeletons and visceral organs, accompanied by clinical symptoms such as loss of appetite, anorexia, the sensation of fullness, weight loss, muscle atrophy, fatigue, anemia, edema, and hypoproteinemia (3). Nevertheless, the diagnosis of cachexia was weight loss greater than 5%, or weight loss greater than 2% in individuals already showing depletion according to current body mass index (BMI < 20 kg/m^2^) or skeletal muscle mass (sarcopenia) (2), and patients are often in the refractory cachexia at the time of diagnosis, making it difficult to effectively control the progression of cachexia. Therefore, it is of great clinical significance to early predict the cancer cachexia and slow down its progress effectively.

Cancer cachexia results from a combination of reduced food intake and metabolic changes, including increased energy expenditure, excessive catabolism, and inflammation (4). Additionally, cancer-induced systemic inflammation could stimulate the release of pro-inflammatory cytokines and increased protein consumption. Systemic inflammation is not only a sign of cachexia, but also a promoter of muscle atrophy (5). A large number of literatures reported that systemic inflammation-related indexes and prognostic-related nutritional indexes were strongly linked to the prognosis of patients (6), including Prognostic Nutritional Index (PNI) (7), Systemic Immune-Inflammation Index (SII) (8), C-reactive protein (9), Serum albumin level (ALB) (10), Serum L-carnitine (11), Neutrophil-Lymphocyte Ratio (NLR) (10), Platelet level (PLT) (12), D-Dimer, and so forth (13). Zhou et al. (14) developed a simple and clinically available cachexia staging score (CSS) for the classification of cachexia stages, whose model showed better discrimination than previous studies and could be used easily in clinical practice. This model facilitates early recognition, diagnosis, and treatment of cachexia. They applied weight loss, decreased appetite, assessment of physical performance, assessment of muscle function and abnormal biochemistry (WBC and hemoglobin and ALB) as the main predictive indicators of the model (14), but no serum nutrition indicators or inflammatory cytokines were involved. Meanwhile, the immune, inflammatory and nutritional factors were greatly associated with the prognosis of cancer (15). However, some studies have exclusively focused on systemic inflammatory markers (such as C-reactive protein and the neutrophil-to-lymphocyte ratio) or solely relied on nutritional indices (such as the Prognostic Nutritional Index, PNI). These single-dimensional models exhibit limitations when dealing with complex clinical scenarios, as they fail to comprehensively reflect the underlying pathophysiological states of patients (16). Additionally, tumor markers, such as Carcinoembryonic Antigen (CEA) and CA 19–9, are well-established prognostic factors and are strongly associated with tumor recurrence in GC patients (17, 18). However, the relationship between tumor markers and cachexia was not unveiled clearly.

Therefore, we aimed to integrate the immunonutrition, inflammatory factors and well-known tumor markers, to construct an immune-inflammatory-nutritional-tumor-marker (IINTM) prognostic score for GC and to study the role of this score in the diagnosis of cachexia, so as to provide assistance for the diagnosis and treatment of patients.

## 2 Methods

### 2.1 Patients and clinical data

A retrospective analysis was conducted on 1,101 gastric cancer patients who underwent radical resection (D1 or D2) between January 2016 and December 2018 at the Department of Digestive Surgery, the First Affiliated Hospital of Air Force Medical University (Xijing Hospital). Initially, 1,172 patients were enrolled in the study; however, we experienced some loss to follow-up during the course of the study, with a final loss to follow-up rate of 6.1%. This resulted in 71 patients not being followed up to the end of the study. To minimize potential biases, we excluded patients who had received neoadjuvant chemotherapy or had active infections, as these factors could significantly influence nutritional and inflammatory markers. Therefore, a total of 1,101 patients with complete medical records, including accurate surgical and pathological reports, were ultimately included in our analysis. All patients were pathologically diagnosed with gastric adenocarcinoma and had complete medical records, including accurate surgical and pathological reports. Blood routine, biochemical indicators, and serum tumor markers were obtained through fasting blood samples at 6 am 1 day before surgery. Nutritional risk screening 2022 (NRS2002) score and BMI were collected before surgery. The tumor stage was assessed using the TNM classification system (Tumor, Node, Metastasis), which describes the extent of cancer based on the size and location of the primary tumor (T), the involvement of regional lymph nodes (N), and the presence of distant metastasis (M). TNM staging was determined according to both clinical diagnosis and postoperative pathological diagnosis.

The inclusion criteria were shown as followed: (1) diagnosis age of GC patients ranged from 18 to 80 years; (2) patients underwent radical resection (D1 or D2); (3) the patients had no blood transfusion, anticoagulation and other relevant treatments that affect the laboratory results; (4) the patients’ medical records were thorough and included accurate surgical and pathological reports. Clear postoperative diagnoses were made, with complete pathological records kept and immunohistochemical analysis conducted; (5) no neoadjuvant chemotherapy was performed before surgery. The exclusion criteria were shown as followed: (1) patients with recurrent gastric cancer; (2) patients with other malignant diseases; (3) preoperative examination indicates that distant metastasis has occurred; (4) patients with missing pathological data; (5) antitumor therapy was performed before surgery.

The follow-up period began after surgery and lasted until December 2020. Regular follow-up visits were scheduled every 3 months during the first 2 years and every 6 months thereafter. Patients were also contacted by telephone if they missed scheduled visits. The median follow-up time for the patients was 47 months. Sensitivity analyses were conducted to assess the potential impact of these lost-to-follow-up patients on the study results, and no significant differences were observed in the overall survival estimates or prognostic model accuracy. Survival time was defined as the duration from the radical resection to the time of death or the last follow-up.

### 2.2 Diagnostic criteria for cachexia

**Figure 1:**
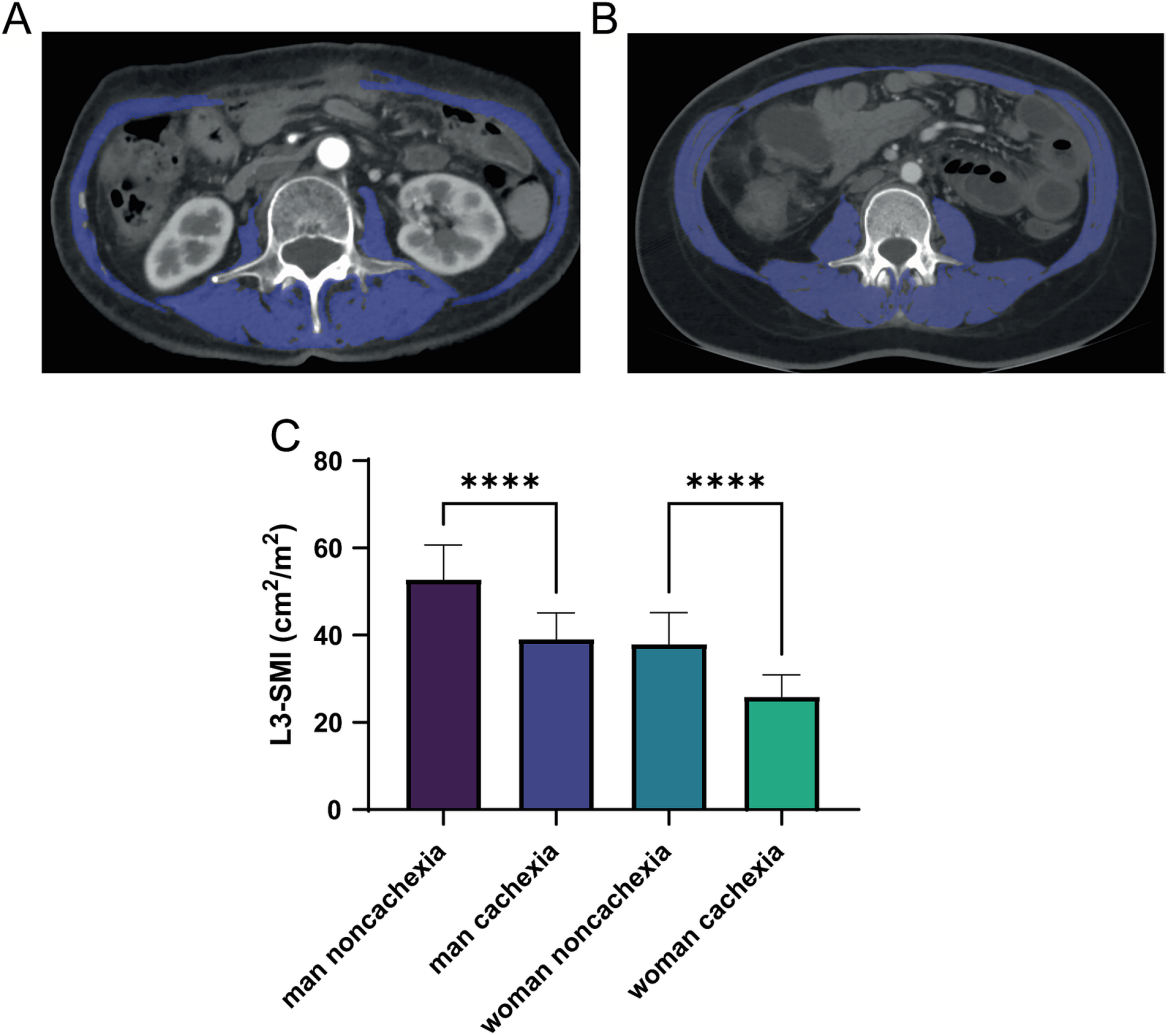
Computed tomography (CT) of low skeletal muscle mass **(A)** and normal **(B)** SMI parameters at L3 levels, with skeletal muscle highlighted in blue; **(C)** differences of L3-SMI parameters among the cachexia and non-cachexia patients with different genders. CT, computed tomography; SMI, skeletal muscle mass; L3, third lumbar spine plane. ****means *p*-values < 0.0001.

The diagnosis of cachexia was weight loss greater than 5%, or weight loss greater than 2% in individuals already showing depletion according to current bodyweight and height (body-mass index <20 kg/m^2^) or skeletal muscle mass (sarcopenia) (2). We validated the skeletal muscle content of normal individuals and cachexia L3 using CT scans, measuring the total area of the third lumbar spine plane skeletal muscle group (mainly including erector spinae, psoas major, psoas quadratus, transversus abdominis, intra-abdominal oblique muscle, external oblique muscle, and rectus abdominis), and calculating its ratio to the square of height, the third lumbar spine plane skeletal muscle mass index (L3-SMI) (cm^2^/m^2^) is obtained (Figure 1).

### 2.3 Evaluation of immunonutrition in patients with GC

The systemic immunonutrition of patients was evaluated by PNI, SII, NLR, ALB, and PLT. PNI consists of ALB and total lymphocyte (TLC) in peripheral blood. SII consists of PLT, absolute neutrophil count (ANC) and TLC. The calculation formula is: PNI = ALB (g/L) + 5 × TLC (10^9^/L) (19). SII = PLT (10^9^/L) × ANC (10^9^/L)/LY (10^9^/L) (20), NLR = ANC (10^9^/L)/TLC (10^9^/L). PLT grade was divided into high-level group (>200 × 10^9^/L) and low-level group (≤200 × 10^9^/L) according to the median of 200 × 10^9^/L.

Subsequently, a restricted cubic spline (RCS) function was applied to reveal the linear or non-linear prognostic patterns of PNI and SII, allowing identification of optimal cutoff values for these indices in GC patients.

### 2.4 Statistical analysis

All statistical analysis was conducted in SPSS 28.0 software. Enumeration data were expressed as constituent ratios or rates (%), and *χ*^2^ test was used for comparison. The median (Interquartile Range, IQR) was used for statistical description, and non-parametric tests were used for comparison between groups. To manage potential outliers in continuous variables, we used IQR analysis. Any data point falling below the first quartile minus 1.5 times the IQR or above the third quartile plus 1.5 times the IQR was considered an outlier. To mitigate the impact of outliers, we applied a winsorizing technique, capping extreme values at the 1st and 99th percentiles. This approach ensures that our analyses are robust and not unduly influenced by extreme values. The survival curves were depicted by the Kaplan–Meier (K–M) curve and further validated by the log-rank test. Multivariable Cox proportional hazards regression analyses were used to construct the prognostic model of GC patients, and the visual nomogram was drawn with the R software ‘rms’ package. The prediction stability of the nomogram was measured by concordance index (C-index) and verified with the calibration curve. The Receiver operating characteristic (ROC) curve and the area under the curve (AUC) were used to verify the diagnostic value of the immunonutrition model for cachexia. *p* < 0.05 was considered as significantly important.

## 3 Results

### 3.1 Distinct immunonutrition and prognostic profiles in cachexia and non-cachexia GC patients

**Figure 2:**
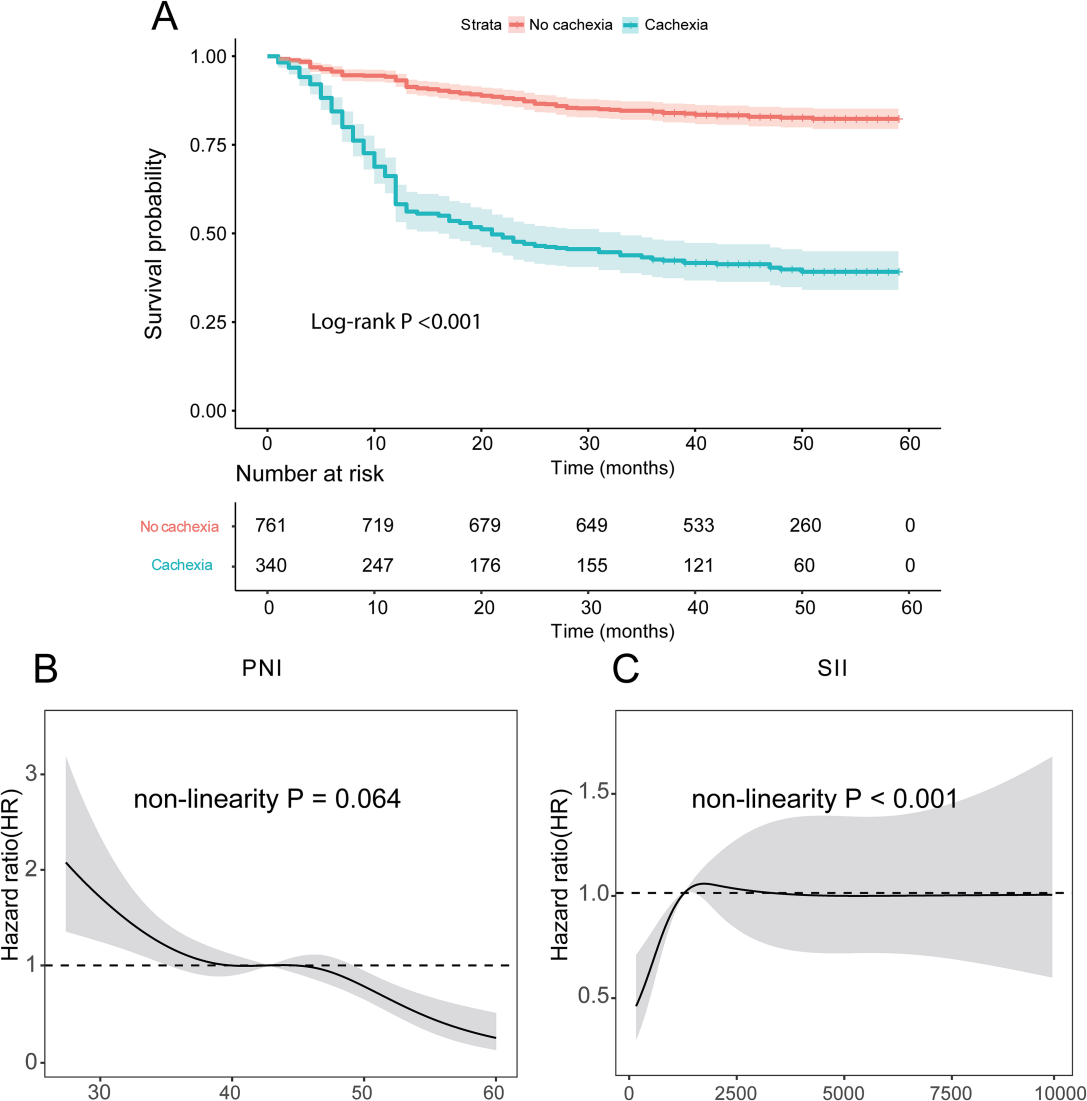
Kaplan–Meier survival curves for GC patients. **(A)** Survival curve of patients with cachexia and non-cachexia; **(B,C)** the relationship of PNI and SII scores with prognosis identified by restricted cubic spline curves. PNI, Prognostic Nutritional Index; SII, Systemic Immune-Inflammation.

This study included 1,101 patients, including 826 males and 275 females, 340 cases of cachexia and 761 with non-cachexia. The median follow-up time of GC patients was 42 months, and the 5-year OS rate was 69.75%. Compared with non-cachexia patients, patients who were later diagnosed with cachexia had lower BMI, lower ALB content, lower lymphocyte count and PNI score, advanced TNM staging, worse Nutritional Risk Screening 2002 (NRS2002 score), higher levels of tumor markers (CEA, CA199 and CA125), higher SII score and D-dimer, prolonger prothrombin time (PT). The difference was statistically significant (*p* < 0.05, Table 1). Additionally, there were remarkably prognostic differences between non-cachexia and cachexia populations, where non-cachexia had overwhelmingly favorable clinical outcomes than cachexia patients (*p* < 0.0001, Figure 2A).

**Table 1:** Baseline features between gastric cancer patients with cachexia and non-cachexia.

Characteristics	Non-cachexia	Cachexia	*p*-value
*N*	761	340	
Gender, *n* (%)			0.539
Male	575 (75.6%)	251 (73.8%)	
Female	186 (24.4%)	89 (26.2%)	
Age, median (IQR)	58 (51, 65)	59 (51.75, 66)	0.371
BMI, median (IQR)	23.597 (21.878, 25.565)	20.434 (18.984, 22.285)	<0.001
TNM, *n* (%)			<0.001
Stage I	300 (39.4%)	17 (5%)	
Stage II	158 (20.8%)	55 (16.2%)	
Stage III	294 (38.6%)	234 (68.8%)	
Stage IV	9 (1.2%)	34 (10.0%)	
NRS2002, *n* (%)			<0.001
<3	274 (36.0%)	83 (24.4%)	
≥3	487 (64.0%)	257 (75.6%)	
ALB, median (IQR)	38.5 (33.9, 43.3)	35.3 (31.075, 39.6)	<0.001
AFP, median (IQR)	2.725 (1.97, 3.84)	2.8 (2, 4.21)	0.239
CEA, median (IQR)	2.05 (1.27, 3.16)	2.97 (1.74, 7.6525)	<0.001
CA199, median (IQR)	9.25 (5.54, 15.98)	35.19 (12.332, 54.8)	<0.001
CA125, median (IQR)	10.67 (7.86, 14.745)	13.395 (9.0725, 21.188)	<0.001
WBC, median (IQR)	7.46 (5.55, 10.98)	8.125 (5.7525, 10.932)	0.395
ANC, median (IQR)	5.69 (3.4993, 9.7523)	6.8472 (4.0932, 9.7857)	0.090
TLC, median (IQR)	1.0906 (0.672, 1.6574)	0.88991 (0.55632, 1.3426)	<0.001
PNI, median (IQR)	44.391 (38.443, 50.847)	39.623 (35.09, 45.807)	<0.001
SII, median (IQR)	1037.7 (447.7, 2514.9)	1,594 (723.99, 2,834)	<0.001
NLR, median (IQR)	5.7445 (2.2327, 14.015)	8.9635 (3.2483, 14.235)	<0.001
PLT, median (IQR)	200 (161, 246)	200.5 (151, 258)	0.444
D-Dimer, median (IQR)	280 (7.11, 575)	370 (177.5, 1052.5)	<0.001
APTT, median (IQR)	34.7 (30.3, 37.6)	34.7 (31.375, 38.3)	0.109
PT, median (IQR)	12.9 (12, 13.6)	13.2 (12.4, 13.8)	<0.001

BMI, body mass index; NRS2002, Nutritional Risk Screening 2002; ALB: Albumin; WBC, white blood cell; ANC, absolute neutrophil count; TLC, total lymphocyte; PNI, Prognostic Nutritional Index; SII, Systemic Immune-Inflammation Index; NLR, Neutrophil-Lymphocyte Ratio; PLT, Platelet; APTT, Activated Partial Thromboplastin Time; PT, Prothrombin Time; IQR: interquartile range.

These results suggest that patients with cachexia have a less immune component, poorer nutritional status, more severe inflammation and worse prognosis.

### 3.2 Exploring PNI and SII as key immunonutrition indicators in GC prognosis

Given that immunonutrition indicators are important factors affecting cachexia and prognosis in patients with GC, and PNI and SII are currently two important and new immunonutrition indicators, this study focused on exploring the impact of PNI and SII on the prognosis of patients with GC. According to the RCS curves, we found that there was a non-linear relationship between overall survival (OS) and neither PNI nor SII indexes (*p* = 0.044 for PNI, Figure 2B; *p* < 0.001 for SII, Figure 2C). When the HR reach 1, the optimal cutoff values were determined for PNI (42.78) and SII (1281.88). Thus, we divided patients into high PNI group (>42.78) and low PNI group (≤42.78), high SII group (>1281.88) and low SII group (≤1281.88).

Furthermore, taking into account the potential prognostic significance of other immunonutrition-related indicators, we conducted supplementary RCS analyses for the NLR and PLT. The RCS analysis demonstrated a non-linear relationship between NLR and Overall Survival (OS) (Supplementary Figure 1). The curve exhibited a non-linear association, where the Hazard Ratio (HR) rapidly increased at lower NLR values, peaked, and then declined, eventually plateauing at higher NLR values. This U-shaped relationship suggests that NLR values within a certain range may correlate with poorer prognosis, whereas at higher NLR values, this correlation diminishes. Similarly, the RCS analysis of PLT also revealed a non-linear association with OS (Supplementary Figure 2), with the HR decreasing at lower PLT values, reaching a nadir, and then increasing as PLT values rise. Given the fluctuation of the curve on both sides of the HR = 1 line, no definitive cutoff value can be ascertained. This indicates that when evaluating NLR and PLT as prognostic indicators, it is necessary to consider their impact across the entire range of values.

Further, we explored whether PNI and SII were significantly affected by age, sex or BMI. To this end, we plotted the relationship between PNI and SII values and age, sex and BMI to assess the potential bias of these factors on PNI and SII. The results indicate that the relationships between PNI and SII and age, BMI are not significant in individuals of different genders (Supplementary Figures 3A–D). After re-adjusting for sex, there were also significant differences in prognosis between the PNI and SII groups. Stratified survival analysis indicated that the survival rate of the high PNI group was higher than that of the low PNI group, and this difference existed in individuals of different genders (Supplementary Figure 3E). The survival rate of the high SII group was lower than that of the low SII group, and this difference existed in individuals of different genders. This indicates that PNI and SII may be independent prognostic factors, but their relationships with age, gender and BMI are relatively weak (Supplementary Figure 3F).

**Figure 3:**
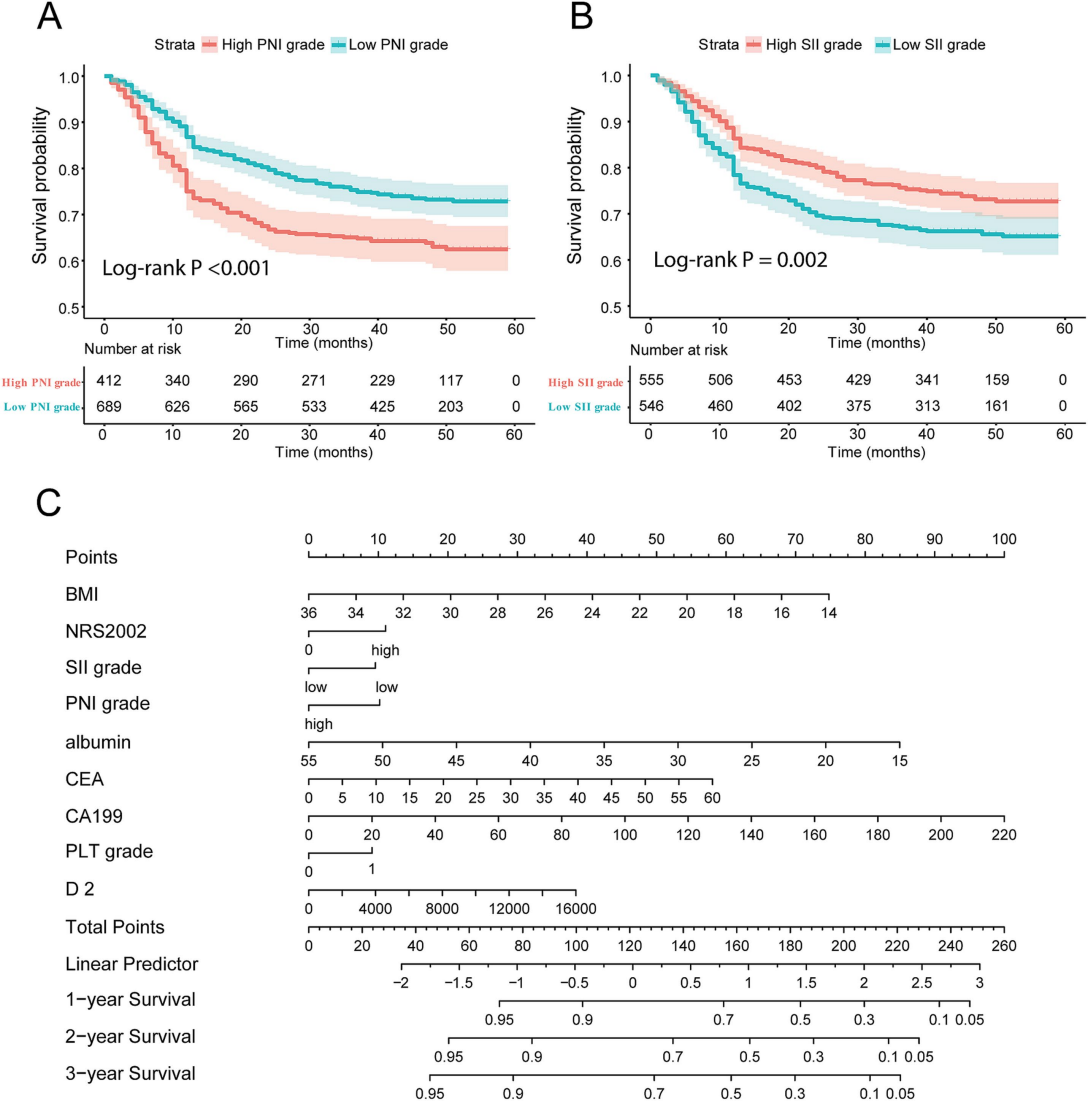
Construction of nomogram composed of immune-nutrition index and tumor biomarker. **(A,B)** Survival curve of patients with different PNI score and SII score grades; **(C)** the nomogram based on immune-nutrition index and tumor biomarkers. PNI, Prognostic Nutritional Index; SII, Systemic Immune-Inflammation.

According to the K-M curve, the 5-year survival rate of the high PNI group was 76.16%, and that of the low PNI group was 63.78%, and the difference was statistically significant (*p* < 0.001, Figure 3A). The 5-year survival rate of the high SII group was 68.80%, and that of the low SII group was 72.50%, with a statistically significant difference (*p* = 0.006, Figure 3B). Taken together, these findings highlight clinical significance of PNI and SII indexes as crucial prognostic factors in GC.

### 3.3 Identification of prognostic factors in GC using univariate and multivariate cox regression analysis

In this study, the clinical characteristics related to the prognosis of GC were analyzed by univariate Cox regression and we found that: Age (hazard ratio, HR = 1.011), BMI (HR = 0.893), NRS2002 score (HR = 1.549), ALB (HR = 0.944), CEA (HR = 1.040), CA199 (HR = 1.012), NLR (HR = 1.010), PNI (HR = 1.715), SII (HR = 0.702), PLT grade (HR = 1.002), D-dimer (HR = 1.051) and PT (HR = 1.091) were associated with prognosis of GC patients (Table 2). The results of univariate Cox regression analysis were incorporated into the multivariate Cox regression analysis, and we found that PNI, SII, BMI, CEA, CA199, and serum ALB were independent factors for the prognosis of GC (*p* < 0.05). In addition, NRS2002 score, PLT, and D-dimer may be closely associated with the prognosis of GC (Table 2). To construct the prognostic score for GC, this study employed a clinical model based on multivariate Cox analysis and utilized the stepwise regression method.

**Table 2:** Cox regression analysis of prognosis in gastric cancer patients.

Characteristics	Total (*N*)	Univariate analysis		Multivariate analysis	
		Hazard ratio (95% CI)	*P*-value	Hazard ratio (95% CI)	*P*-value
Gender	1,101		0.898		
Male	826	Reference			
Female	275	1.016 (0.794–1.301)			
Age	1,101	1.011 (1.001–1.021)	0.036	1.001 (0.991–1.012)	0.785
BMI	1,101	0.893 (0.862–0.926)	<0.001	0.923 (0.889–0.958)	<0.001
NRS2002	1,101		<0.001		0.055
<3	357	Reference		Reference	
≥3	744	1.549 (1.207–1.988)		1.286 (0.993–1.666)	
ALB	1,101	0.944 (0.927–0.961)	<0.001	0.953 (0.925–0.981)	0.001
AFP	1,085	1.003 (0.999–1.007)	0.152		
CEA	1,101	1.040 (1.030–1.050)	<0.001	1.022 (1.011–1.034)	<0.001
CA199	1,101	1.012 (1.010–1.015)	<0.001	1.010 (1.008–1.013)	<0.001
CA125	1,097	1.001 (1.000–1.002)	0.081		
WBC	1,101	1.010 (0.983–1.038)	0.483		
NLR	1,101	1.010 (1.001–1.021)	0.0458	1.001 (0.987–1.016)	0.8122
PNI	1,101		<0.001		0.032
≤42.78	551	Reference		Reference	
>42.78	550	1.715 (1.378–2.137)		1.576 (1.081–2.298)	
SII	1,101		0.001		0.019
≤1281.88	551	Reference		Reference	
>1281.88	550	0.702 (0.566–0.873)		0.823 (0.703–0.964)	
PLT-grade	1,101		0.012		0.067
≤200	545	Reference		Reference	
>200	556	1.002 (1.000–1.003)		1.234 (0.983–1.548)	
D-Dimer	1,099	1.051 (1.005–1.099)	<0.001	1.062 (0.994–1.135)	0.073
APTT	1,100	1.005 (0.987–1.024)	0.566		
PT	1,100	1.091 (1.004–1.186)	0.040	0.987 (0.900–1.083)	0.783

CI, confidential interval; BMI, body mass index; NRS2002, Nutritional Risk Screening 2002; ALB, albumin; WBC, white blood cell; NLR, neutrophil-lymphocyte ratio; PNI, Prognostic Nutritional Index; SII, Systemic Immune-Inflammation Index; APTT, activated partial thromboplastin time; PT, prothrombin time.

### 3.4 Constructing and validating the IINTM prognostic score and nomogram in GC patients

According to the multivariate Cox regression, the prognostic IINTM score was constructed by stepwise regression method: IINTM score = 0.0103 × PNI − 0.254 × SII – 0.0764 × BMI + 0.2699 × NRS2002–0.0418 × ALB − 0.0225 × CEA + 0.01022 × CA199 + 0.2541 × PLT grade + 0.0387 × D-dimer. The C-index of the IINTM score was 0.784, which indicated that the prediction accuracy was satisfactory.

Next, we established a nomogram that could be used clinically by multiple prognostic factors, comprised of BMI, NRS2002, SII grade, PNI grade, ALB, CEA, CA199, PLT grade and D-dimer. The precise values for each variable were extracted from the nomogram, and their summation equated to the total points. Finally, the 1-year, 2-year, and 3-year survival rate was predicted according to the total points which are located on the corresponding axes (Figure 3C).

### 3.5 Calibration and validation of the nomogram for predicting survival in GC

**Figure 4:**
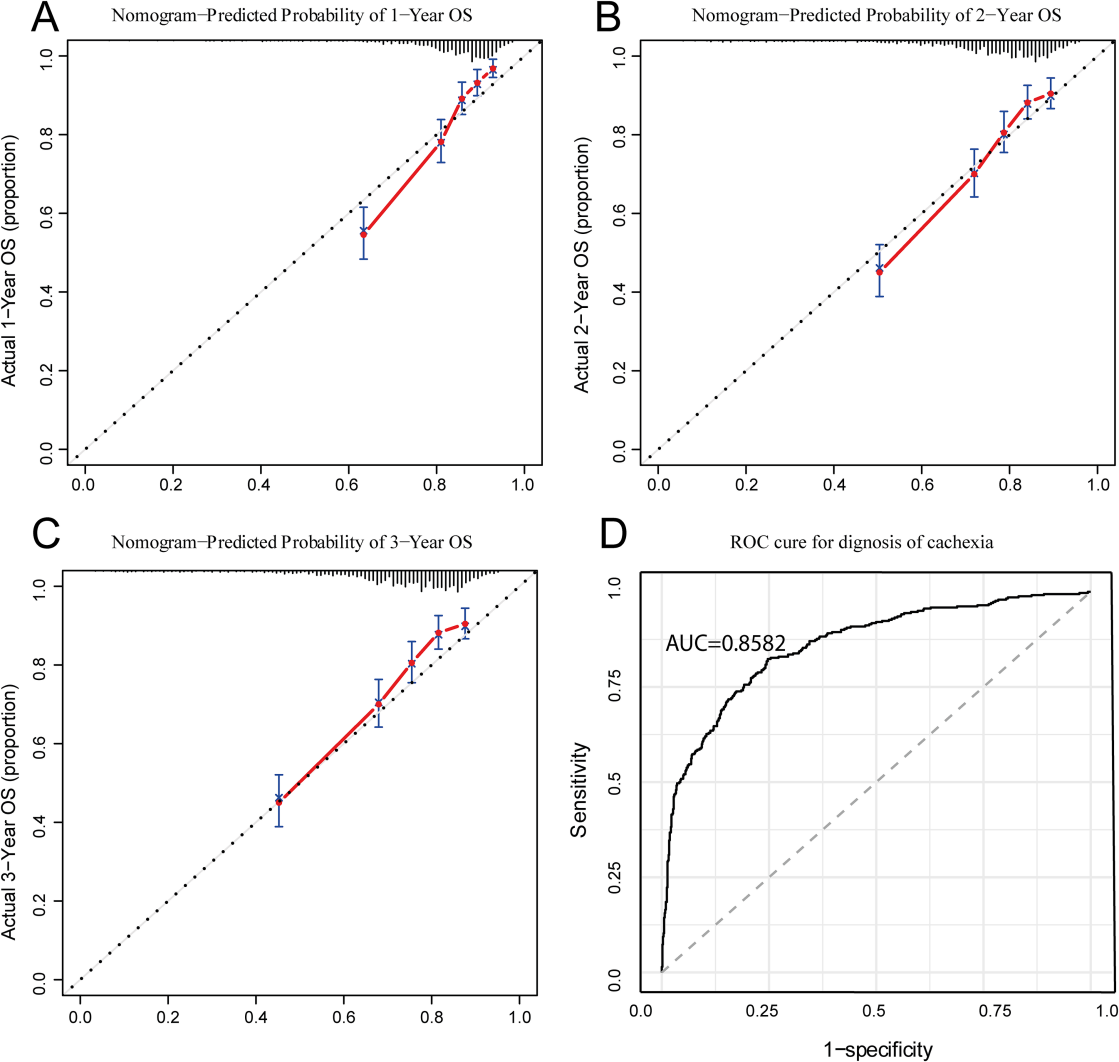
Calibration and ROC curves for IINTM score model. **(A–C)** Calibration curves of IINTM for predicting 1-, 2-, and 3- year OS; **(D)** ROC curve of the IINTM score model for cachexia. IINTM, immune-inflammatory-nutritional-tumor-marker; OS, overall survival; ROC, receiver operating characteristic.

In order to verify the consistency of the prediction of the nomogram, this study drew the calibration curve and uses Bootstrap resampling 1,000 times to draw the calibration curve to verify the established prediction model. The *X*-axis of the calibration curve represents the survival rate predicted by the nomogram, and the *Y*-axis represents the actual survival rate of the patients. The accuracy of the nomogram is reflected by the fitting of the solid lines and the dotted lines. The results showed that the 1-year, 2-year, and 3-year survival rates of our study had good coincidence between the observed values of the calibration curve and the predicted values of the nomogram (Figures 4A–C).

### 3.6 Evaluating the diagnostic accuracy of the IINTM score for cachexia in GC patients

Because this IINTM score was mainly composed of systemic immunity, nutrition-related and tumor marker features, we speculated that this score system may be strongly correlated with the diagnosis of cachexia. Therefore, we investigated the relationship of IINTM score with cachexia, that is, to evaluate the diagnostic efficiency of the score for cachexia. As anticipated, the Area Under the Receiver Operating Characteristic Curve (AUC) was found to be 0.858, indicating that the IINTM score can accurately predict the early onset of cachexia in patients (Figure 4D). The above results show that the IINTM composed of systemic inflammatory indicators and nutritional indicators, BMI, ALB and tumor markers (CEA, CA199) could also accurately predict the state of GC cachexia.

## 4 Discussion

This study found that immunonutrition was closely associated with clinical outcomes of GC patients. Serum ALB, PNI index, SII index, platelet count, and BMI were closely related to the prognosis of GC, and IINTM score comprising these indicators demonstrated a good accuracy in diagnosing cachexia. Finally, the nomogram constructed based on the IINTM in this study is simple and accurate and can provide important significance for clinical decision-making.

Cachexia is the result of malnutrition-related chronic diseases such as cancer, chronic heart failure, chronic renal failure, autoimmune diseases. Among them, cancer is the most common cause of cachexia (3). Clinically, nutritional supplementation alone cannot improve the process of cachexia, due to the highly expressed cellular pro-inflammatory factors and enhanced catabolism caused by tumors. Cancer cachexia could also reduce the efficacy of chemotherapy, increase its side effects and lead to discontinuation of treatment and even poor survival (21–23). Early detection and timely diagnosis of cachexia is crucial to the reversal of malignant nutritional status and prolongation of survival in cancer patients. Additionally, a great deal of literatures indicated that the systemic inflammation and extremely poor nutritional status of patients were not only highly related to cachexia, but also closely related to the prognosis of patients (24, 25). Therefore, the purpose of this study is to construct a prognostic score system based on systemic immune, nutritional status and tumor marker, and to evaluate the diagnostic value of this score for cachexia.

First, this study examined 1,101 patients diagnosed with gastric adenocarcinoma, including patients diagnosed with cachexia and non-cachexia. The baseline features revealed that patients with cachexia were characterized by significantly decreased levels of immune cells, serum protein, basic BMI, and NRS2002 scores. This implies that the status of immunonutrition and associated parameters significantly influence the progression of GC patients toward cachexia. Moreover, the cachexia population had worse prognosis than non-cachexia patients. Next, we evaluated the value of immunonutrition status in the prognosis of patients with GC. By employing univariate and multivariate Cox regression analyses, and utilizing the stepwise regression approach, we formulated the IINTM scoring system specifically for patients who underwent radical resection for gastric cancer. The resulting prognostic value proved significant, demonstrating a C-index of 0.784, indicative of exceptional stability. When studying the impact of individual immunonutrition indicators on prognosis, we focused on the novel comprehensive indicators: PNI and SII scores. According to the K-M survival curve, patients with lower levels of PNI had poorer prognosis, while patients with lower levels of SII had better prognosis. PNI is a parameter composed of serum ALB and total lymphocytes, which represents the systemic and immune status. As reported in most literatures, the lower PNI level, the worse the prognosis (26–28). SII is the product of platelets and NLR and represents the systemic inflammatory state of the patient. With a low level of inflammatory state, the probability of cachexia in patients is low. Recently, it has been reported in the literature that the AUC of SII for the prediction of cachexia in locally advanced GC can reach 0.930, which is better than that of PNI for the prediction of cachexia (28). At the same time, in highly malignant pancreatic ductal cell carcinoma, the incidence of cachexia remains high, and the higher the expression of SII, the worse the prognosis of the patients (29). This study explored the impact of SII and PNI on OS in a large sample of GC patients, fully demonstrating the impact of immune-inflammatory-nutrition on GC patients’ prognosis.

Then, we calculated the patient’s IINTM prognosis score through multivariate Cox regression analysis and stepwise regression method, and we found that tumor markers (CEA, CA199) played a considerable role in the score system. The continuous increase of CEA indicates the risk of recurrence after surgery, and it has been reported in the literature that circulating CEA-positive tumor cells can be used as a marker of GC recurrence (30). Similarly, the expression of serum CA199 can also indicate GC recurrence and is associated with poor prognosis (31). However, there was little research focus on the relationship between CEA, CA199 and cachexia, and the relevant results need further verification. This IINTM score included indicators such as tumor markers, which could predict the prognosis more accurately. Meanwhile, this study found that the IINTM composed of immunonutrition and tumor markers has a good predictive value for the diagnosis of cachexia, and the AUC reaches 0.85. The IINTM score is designed to provide an early indication of cachexia risk, facilitating timely intervention and management. Early detection of cachexia risk can prompt earlier nutritional intervention, helping to maintain muscle mass and improve quality of life. Additionally, the IINTM score can inform treatment decisions, guiding the implementation of more aggressive supportive care measures for high-risk patients. This tailored approach can lead to better patient outcomes and improved quality of life. Additionally, the IINTM score provides clinicians with a quantitative tool to assess patient risk and prognosis, supporting more informed decision-making regarding treatment options. While our study focused on gastric cancer patients, we believe that the principles of integrating immune-inflammatory, nutritional, and tumor marker parameters can be extended to other cancer types with appropriate modifications. The above results show that immunonutrition-related indicators and tumor markers could not only accurately predict the prognosis of patients with GC, but also have considerable value in the prediction of cachexia. In order to further promote clinical application, we developed a visual nomogram in this study. The nomogram designed based on the above immunonutrition indicators could help clinical decision-making and provide a reference for the prediction of cachexia. The simplicity and clinical feasibility of cachexia diagnosis make it a promising tool for predicting tumor survival. This study demonstrates that cachexia, as defined by the criteria used in our research, is strongly associated with poor prognosis in gastric cancer patients. This finding suggests that cachexia can serve as a clinically relevant and easily assessable prognostic indicator. Cachexia often precedes significant tumor progression and can be detected early in the disease course. By identifying patients who develop cachexia, clinicians can proactively implement supportive care measures and consider more aggressive treatment strategies to potentially improve outcomes.

However, there are several limits in our study. First, despite the relatively large sample size (*n* = 1,101), the study was conducted in a single center, and the results only reflect the characteristics of the GC population in Northwest China. Therefore, the representativeness of our sample across different regions and races cannot be fully confirmed. Given this limitation, there is an urgent need for multicenter prospective studies to consolidate our findings and to better understand the generalizability of our results to other populations. Second, PNI and SII have poor ability in predicting cachexia, but have a greater value in prognosis. Thirdly, some other potential confounders which could affect prognosis could not be considered in the study.

Cachexia is one of the most serious complications in patients with GC. While cachexia is indeed a symptom, it is also a significant prognostic indicator and a critical factor in the overall management of cancer patients. Patients with cachexia have a very poor prognosis and low quality of life. This study was based on the clinical differences between cachexia and non-cachexia patients, and then studied the impact of inflammation and immune indicators on the prognosis of GC and constructs a novel IINTM score integrated with GC tumor markers. The IINTM score is not intended to replace established clinical judgment but rather to augment it by providing a quantitative and comprehensive assessment of patient risk. By identifying patients at high risk of developing cachexia, the IINTM score can facilitate earlier and more targeted interventions, potentially improving patient outcomes. This prognostic score could not only reflect the prognosis and immunonutrition state of patients with GC, but also accurately predict the probability of cachexia in the later stage of the patient, providing basis and guidance for early detection and effective treatment of cachexia.

## Data Availability

The original contributions presented in the study are included in the article/Supplementary material, further inquiries can be directed to the corresponding authors.

## Supplementary material

SUPPLEMENTARY FIGURE 1 The relationship of NLR with prognosis identified by restricted cubic spline curves.

SUPPLEMENTARY FIGURE 2 The relationship of PLT with prognosis identified by restricted cubic spline curves.

SUPPLEMENTARY FIGURE 3 The relationship between PNI and SII and age, gender, BMI and survival rate. **(A,B)** The relationship between PNI and SII and age and gender; **(C,D)** the relationship between PNI and SII and BMI. **(E,F)** The survival analyses of the relationship between PNI and SII and survival rate after re-adjusting for gender.
