# Supplementary figures and images for: Prognostic value of combining preoperative immune-inflammatory-nutritional index and tumor biomarkers in gastric cancer patients undergoing radical resection
Source: Front Nutr. 2025 Jun 10;12:1562202. doi: 10.3389/fnut.2025.1562202 (PMC12185276; doi:10.3389/fnut.2025.1562202)

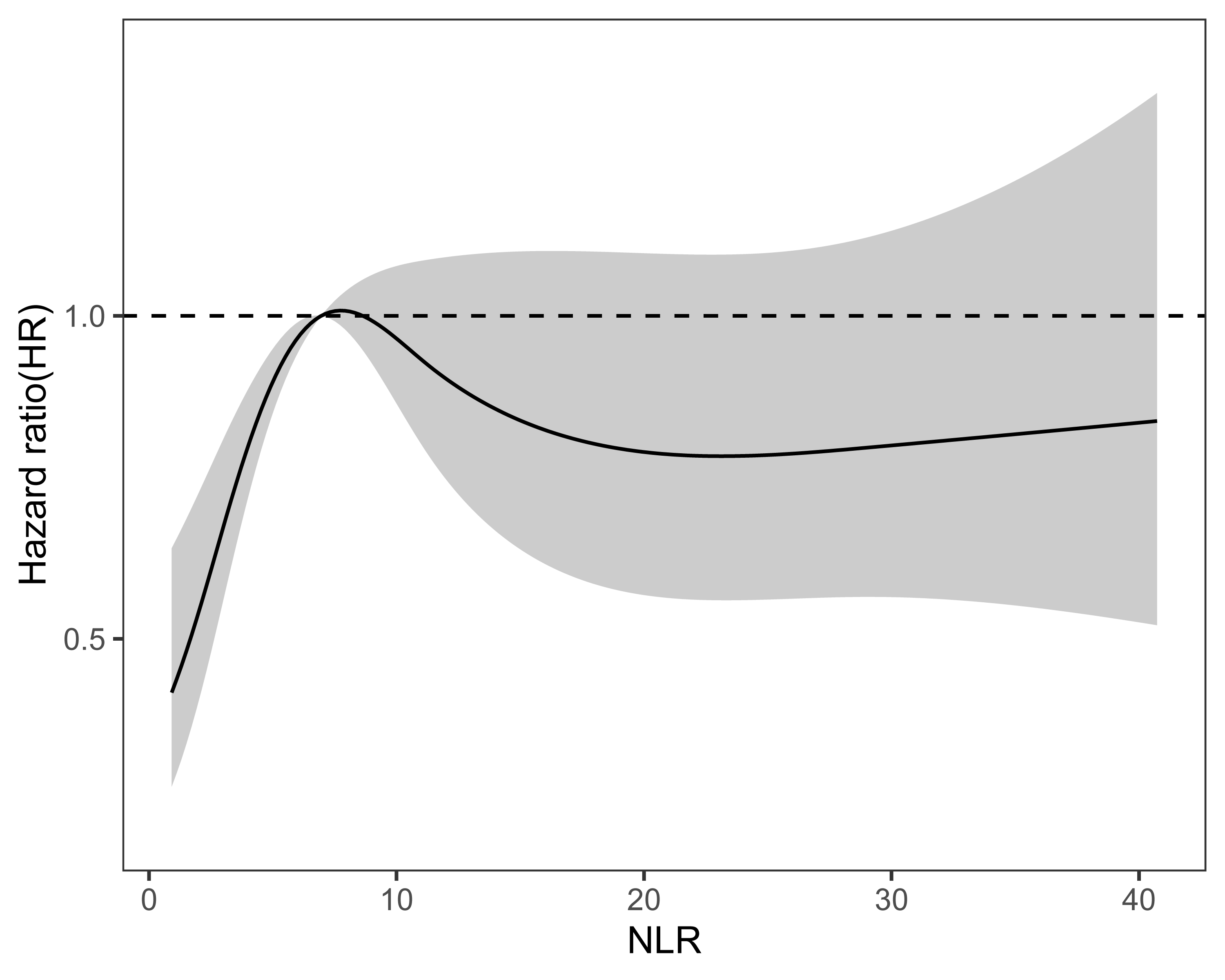

Supplement: SUPPLEMENTARY FIGURE 1 — The relationship of NLR with prognosis identified by restricted cubic spline curves. [file Image_1.tif]

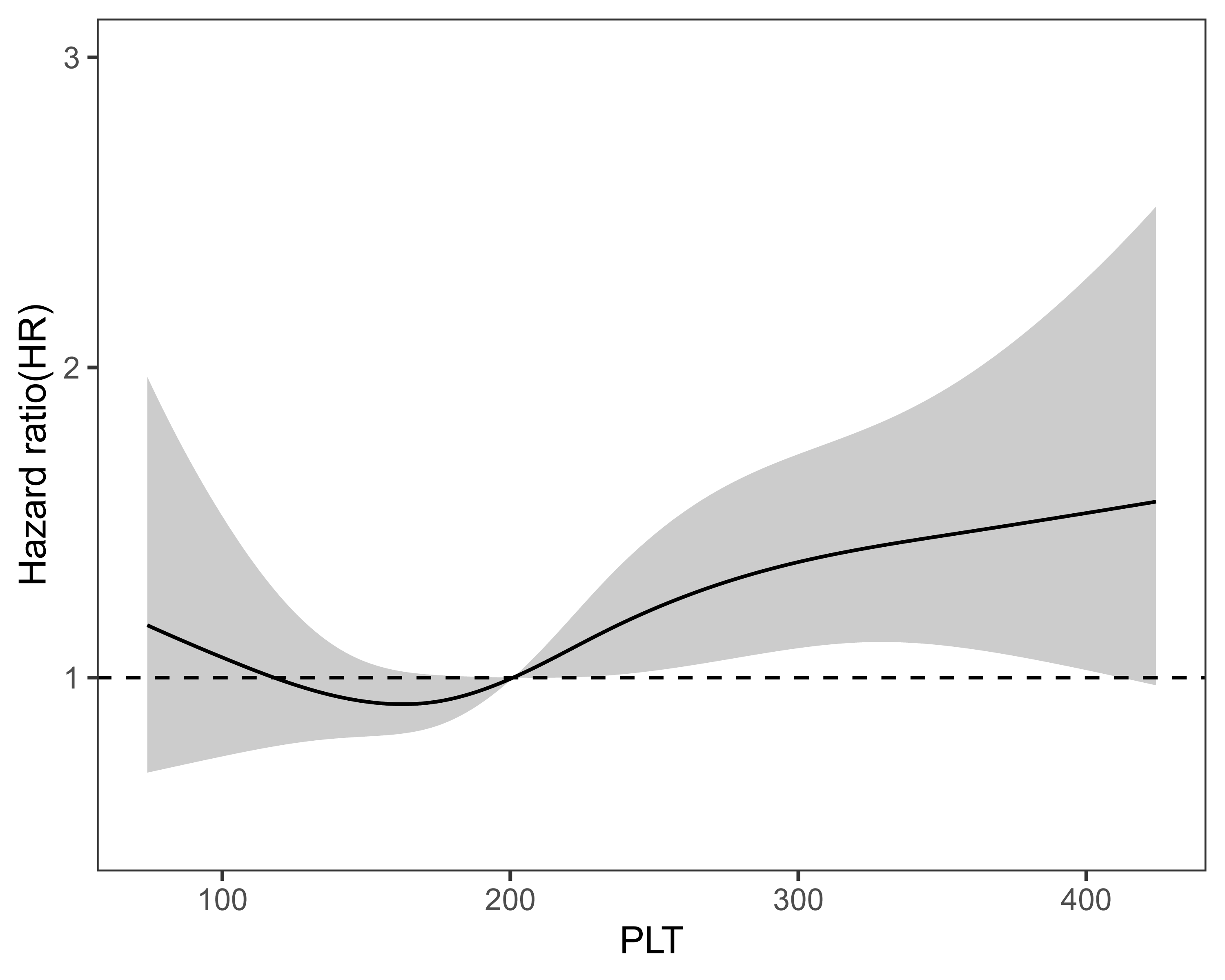

Supplement: SUPPLEMENTARY FIGURE 2 — The relationship of PLT with prognosis identified by restricted cubic spline curves. [file Image_2.tif]
